# Alum as a Remedy in Croup

**Published:** 1857-07

**Authors:** 


*Alum as a Remedy in Croup.*—A correspondent of the New Hampshire Journal of Medicine states that for three years he has used alum in croup, and in all that time has not seen a fatal case which was treated with it from the begining. He usually gives about ten grains, once in ten minutes, until vomiting is induced, using at the same time tartar emetic or the hive syrup freely—the latter subduing the inflamation, while alum has more of a repulsive action.—*St. Med. and Sur. Journal*

